# *Haematococcus pluvialis* Accumulated Lipid and Astaxanthin in a Moderate and Sustainable Way by the Self-Protection Mechanism of Salicylic Acid Under Sodium Acetate Stress

**DOI:** 10.3389/fpls.2021.763742

**Published:** 2021-11-19

**Authors:** Qunju Hu, Mingjian Song, Danqiong Huang, Zhangli Hu, Yan Wu, Chaogang Wang

**Affiliations:** ^1^Shenzhen Key Laboratory of Marine Bioresource and Eco-Environmental Science, Shenzhen Engineering Laboratory for Marine Algal Biotechnology, Guangdong Provincial Key Laboratory for Plant Epigenetics, College of Life Sciences and Oceanography, Shenzhen University, Shenzhen, China; ^2^Marine Resources Big Data Center of South China Sea, Southern Marine Science and Engineering Guangdong Laboratory Zhanjiang, Zhanjiang, China; ^3^Instrumental Analysis Center, Shenzhen University, Shenzhen, China

**Keywords:** salicylic acid and sodium acetate stresses, *Haematococcus pluvialis*, transcriptomic analysis, metabolic coordination, astaxanthin biosynthesis, fatty acids biosynthesis

## Abstract

To elucidate the mechanism underlying increased fatty acid and astaxanthin accumulation in *Haematococcus pluvialis*, transcriptome analysis was performed to gain insights into the multiple defensive systems elicited by salicylic acid combined with sodium acetate (SAHS) stresses with a time course. Totally, 112,886 unigenes and 61,323 non-repeat genes were identified, and genes involved in carbon metabolism, primary and secondary metabolism, and immune system responses were identified. The results revealed that SA and NaAC provide both energy and precursors to improve cell growth of *H. pluvialis* and enhance carbon assimilation, astaxanthin, and fatty acids production in this microalga with an effective mechanism. Interestingly, SA was considered to play an important role in lowering transcriptional activity of the fatty acid and astaxanthin biosynthesis genes through self-protection metabolism in *H. pluvialis*, leading to its adaption to HS stress and finally avoiding massive cell death. Moreover, positive correlations between 15 key genes involved in astaxanthin and fatty acid biosynthesis pathways were found, revealing cooperative relation between these pathways at the transcription level. These results not only enriched our knowledge of the astaxanthin accumulation mechanism in *H. pluvialis* but also provided a new view on increasing astaxanthin production in *H. pluvialis* by a moderate and sustainable way in the future.

## Introduction

Astaxanthin (C_40_H_52_O_4_, 3, 3′-dihydroxy-β, β-carotene-4, 4′-dione) is a bright red secondary carotenoid from the xanthophylls group, which is considered to be the most potent antioxidant in nature, and an important pigment for industrial markets (Machado et al., 2016; Shah et al., 2016; Molino et al., 2018). Naturally, astaxanthin is obtained from aquatic and non-aquatic microorganisms, such as bacteria, yeasts, fungi, and microalgae (Silva et al., 2012; Molino et al., 2018; Wan et al., 2021). *Haematococcus pluvialis*, a unicellular microalga distributed in various habitats worldwide, is considered the best astaxanthin natural source (Ranga Rao et al., 2010). The life cycle of this microalga consists of four phases, including macrozooid (zoospore), microzooids, palmella, and aplanospore (Shah et al., 2016). Stress conditions, e.g., nutrition deficiency, ferrous ion addition, salinity, high light intensity, UV-B irradiation, and drought, induce astaxanthin biosynthesis in *H. pluvialis* cells from lower content, 0.1% on a dry basis, in the macrozooid phase until the range of 1.5–4% on a dry basis in the aplanospore phase (Fábregas et al., 2003; Cerón et al., 2007; Chen et al., 2015; Hong et al., 2015; Hu et al., 2020). Various plant hormones and their derivatives can also affect astaxanthin accumulation in *H. pluvialis* (Shah et al., 2016). Under stress conditions, *H. pluvialis* cells also accumulate large amounts of neutral lipids in the aplanospore phase. Approximately, 96% of astaxanthin molecules are predominantly esterified with fatty acids and deposited in triacylglycerols (TAG)-rich cytosolic lipid globules (LBs). Thus, the increased neutral lipids in the cells are hypothesized to be a matrix for solubilizing of the esterified astaxanthin, and the suitable profile of lipids indicates a possibility of biodiesel production from *H. pluvialis* (Holtin et al., 2009; Saha et al., 2013; Shah et al., 2016). Although *H. pluvialis* is the best natural source of astaxanthin known to date, its productivity still needs to be improved. Furthermore, the ability to produce additional high-value compounds in addition to astaxanthin will enhance economic value of *H. pluvialis* (Le-Feuvre et al., 2020).

Various carbon sources have been studied for mixotrophic or heterotrophic cultivation of *H. pluvialis*, including acetate, glucose, glycerol, rice straw hydrolyzates, and organic-rich wastewaters (Leite et al., 2015; Gupta et al., 2019). *H. pluvialis* can also utilize exogenous organic carbon substrates (Kobayashi et al., 1992), and sodium acetate (NaAC) was considered to significantly accelerate cell growth or astaxanthin accumulation (Kobayashi et al., 1993; Orosa et al., 2001; Tolga et al., 2010; Pang et al., 2019; Zhang et al., 2019; Cong et al., 2020). In *H. pluvialis*, exogenous NaAC increases respiratory rates and suppresses photosynthetic activity in the cells (Zhang et al., 2019). It can be used as an effective carbon source, providing energy for the biosynthesis of astaxanthin and fatty acids (Cong et al., 2020; Oslan et al., 2021; Wang et al., 2021; Zhao et al., 2021). NaAC can be utilized by the tricarboxylic acid cycle to control the flux of carbon. It is postulated that NaAC enhances astaxanthin accumulation in *H. pluvialis* cells in three ways, including, (i) Increased acetyl-CoA from the carbon flux directly enhances respiratory rates of the cells and provides both NAD(P)H and carbon skeletons for the biosynthesis of astaxanthin; (ii) Increased acetyl-CoA indirectly enhances biosynthesis of fatty acids and accelerates the accumulation of astaxanthin; (iii) Enhanced photoprotection capacity promotes the posttranslational activation of carotenoid biosynthesis and fatty acid elongation (Kobayashi et al., 1993; He et al., 2018; Zhang et al., 2019).

Li et al. (2020) indicated that the effective strategy to enhance astaxanthin production was to apply stress to induce astaxanthin production through directing the flow and partition of carbon toward lipids and astaxanthin biosynthesis while maintaining biomass growth by keeping carbon fixation and assimilation at the same time. Biomass growth rates of microalgae could be determined roughly by organic carbon uptake and photosynthesis rates (Li et al., 2020). Astaxanthin accumulated in *H. pluvialis* cells has been known to be deposited in cytoplasm lipid droplets (Pick et al., 2019). The increasing titer of lipids in the *Haematococcus* biomass was considered to increase the astaxanthin content simultaneously. Overexpression of the relevant genes that encode the esterifying enzymes might promote lipid and astaxanthin biosynthesis (Li et al., 2020). However, the effect of NaAC on the accumulation of astaxanthin is always integrated with the increase of biomass production (Göksan et al., 2010). Transcriptome analysis demonstrated that the addition of NaAC under high light condition upregulated several pathways of basal and secondary metabolism, including carbon fixation in photosynthetic organisms, fatty acid elongation, biosynthesis of unsaturated fatty acids, and carotenoid biosynthesis, resulting in enhanced astaxanthin accumulation (Hu et al., 2019). However, adding an excessively high concentration of NaAC (HS) has increased the damage to *H. pluvialis* cells in our pretest study.

In addition, chemicals are used as effective and economic stimulants to promote astaxanthin content in *H. pluvialis* cells (Yu et al., 2015; Cui D. et al., 2020; Li et al., 2020, 2021). Currently, small chemical regulators, especially phytohormones, have been intensively studied for their effects on the physiology and bioproduct biosynthesis in microalgae (Yu et al., 2015; Li et al., 2020). The phytohormones, such as abscisic acid, brassino steroids, diethyl aminoethyl hexanoate, fulvic acid, gibberellins, melatonin, methyl jasmonate, and salicylic acid, have been demonstrated to substantially affect the physiology, biomass growth, or astaxanthin accumulation of the *Haematococcus* cells (Lu et al., 2010; Raman and Ravi, 2011; Gao et al., 2012a, b, 2013a,b; Zhao Y. et al., 2015; Zhao et al., 2018, 2019; Ding et al., 2018a, b, 2019a; Hu et al., 2021). Salicylic acid (SA) is an important phenolic compound involved in regulating the growth and development of plants, and also regulating the responses of plants to biotic and abiotic stress factors (Raskin, 1992; Khan et al., 2010, 2012, 2013, 2014, 2015; Miura and Tada, 2014). SA is considered to significantly regulate some important plant physiological processes, for example, photosynthesis, nitrogen metabolism, proline metabolism, production of glycine betaine, plant water relations, and antioxidant defense system, under stress conditions and thereby protects plants against abiotic stresses (Khan et al., 2010, 2012, 2013, 2014, 2015; Nazar et al., 2015; Miura and Tada, 2014). Fu et al. (2021) revealed that SA, as a signaling molecule, accelerated growth and proliferation of microalgal through exhibited positive impacts on DNA replication, carbon assimilation, and TCA cycle of the cells. SA stimulates astaxanthin accumulation at moderate levels (Lu et al., 2010) and inhibits astaxanthin biosynthesis at high concentrations in *H. pluvialis* (Raman and Ravi, 2011). It was indicated that SA enhanced the transcriptional expression of eight carotenogenic genes in *H. pluvialis*, although these genes exhibited different expression patterns (Gao et al., 2012b). Following supplementation of *H. pluvialis* cultures with SA, a time course-dependent changes in the transcriptome analysis identified several related genes coding for enzymes and transcription factors related to the carotenoid and astaxanthin metabolism (Gao et al., 2015). Gao et al. (2016) also indicated that the proteins enriched in lipid metabolism are regulated differently in SA and JA treatments with time, consistent with the genetictranscriptional expressions of genes involving in the fatty acid biosynthesis pathway.

Transcriptome analysis has become a preferred tool for gene transcription level studies (Cheng et al., 2017). Transcriptome data are valuable resources for investigating plants exposed to stress conditions. Because of the inaccessibility of a complete reference genome sequence to quantifying the transcriptome, global transcriptomic analysis of *H. pluvialis* was slowly investigated. And the potential mechanisms of SA on *H. pluvial is* combined with other environmental stresses in protecting *H. pluvialis* cells against these abiotic stresses, and improving the fatty acid and astaxanthin biosynthesis process is still under question. Thus, we chose SA and HS as variables in this study to explore the molecular mechanism of the *H. pluvialis* of astaxanthin and fatty acid biosynthesis by transcriptome analysis according to SAHS treatment time. Based on the genome database of *H. pluvialis* in our previous report (Luo et al., 2018), detailed genetic information revealed its resistance mechanism. The sequenced unigenes were assembled by the reported genome data and then annotated in various databases, including COG, GO, kyoto encyclopedia of genes and genomes (KEGG), NR, Swiss-Prot, and PFAM. The assembling and the annotation of the transcriptome sequences were considered to provide valuable genomic information for greater insight into the molecular mechanism of SA in *H. pluvialis* in response to HS concentration. The abovementioned results were further supported by the qRT-PCR results, changes in cell morphology and intracellular pigments, and fatty acids contents of *H. pluvialis* cells. In sum, this study provides a new practical method to increase astaxanthin and fatty acids accumulation in *H. pluvialis* and insights into the underlying mechanism promoting SA effect on industrial production of astaxanthin and biodiesel in *H. pluvialis*.

## Materials and Methods

### Microalga Strains and Culturing Conditions

In this study, *Haematococcus pluvialis* 192.80 obtained from Sammlung von Algenkulturen Göttingen Culture Collection of Algae was cultured in Bold Basal Medium media in 250-ml Erlenmeyer flasks with permeable sealing membranes. Cells were cultured at 22°C under continuous fluorescent light (20 μmol⋅m^–2^⋅s^–1^) until the logarithmic phase (about 1 × 10^5^ cells⋅ml^–1^). Then, the cells were collected together and evenly divided into 18 aliquots (300 ml algal culture in each 500 ml Erlenmeyer flasks) and used for salicylic acid (SA) and high concentration of sodium acetate (HS, 45 mM) treatments (SAHS). The SA dosages were at 25 mg⋅L^–1^ for the induction (Gao et al., 2012b, 2015), and cultures were sampled at 0, 1, 6, 12, 24, and 48 h, respectively. The sampled cells were centrifuged at 6,000 *g* for 5 min, harvested and immediately frozen in liquid nitrogen for 10 min, and stored at −80°C until further analysis. Each treatment consisted of three biological repeats.

### Microalgal Morphology

The microalga cells were observed on an Olympus BX61 microscope with an Olympus DP10 digital camera (Olympus, Japan) at each sampling time to track the progress of morphology and color changes of *H. pluvialis* cells during the treatments.

### Growth, Lipid, and Astaxanthin Analysis

Cell numbers were calculated by the Thoma counting method using an Olympus CX40 microscope (Olympus, Japan), and the biomass dry cell weight (DCW) was measured gravimetrically at each sampling time (Hu et al., 2015). Fatty acid methyl ester (FAME) analysis was determined using a method previously optimized by Ma et al. (2018), and astaxanthin yield was measured using spectrophotometry (Boussiba et al., 1992).

### RNA Extraction and cDNA Library Construction

*Haematococcus pluvialis* cells sampled from control, and SAHS treatment at 1, 6, 12, 24, and 48 h (named SAHS_1, SAHS_6, SAHS_12, SAHS_24, and SAHS_48) at the same stages were merged together for the isolation of total RNA. RNA was extracted using TRIzol reagent (Invitrogen, United States) according to the manufacturer’s protocols. The extracted quality of RNA was checked by agarose gel electrophoresis and the BioPhotometer Plus photometer (Eppendorf, Germany). RNA integrity was evaluated using the Agilent 2100 Bioanalyzer (Agilent Technologies, United States), and libraries with RNA integrity ≥5 were subsequently analyzed. Then, the construction, validation, and sequencing of the RNA libraries were conducted using the method mentioned in our previous report in cooperation with Majorbio Co. (Shanghai, China) (Hu et al., 2021).

### Sequence Assembly and Annotation

Raw reads were processed using FASTX-Toolkit^1^. Reads containing more than 5% unknown nucleotides and the low-quality reads were removed using SeqPrep^2^ and Sickle^3^ to obtain clean reads, and further mapped to the reference genome using TopHat2^4^. Subsequently, the mapped reads were assembled using the TopHat-Cufflinks platform^5^, and extension of gene structure and identification of novel transcripts were performed through comparison with reference genome and known annotated genes using the Cuffcompare software (Trapnell et al., 2010). Following assembly of the reads, annotation of all these unigenes was processed using BLAST with a cutoff *E*-value of e^–5^ based on various protein databases, including COG (Clusters of Orthologous Groups of proteins), GO (Gene Ontology), KEGG, NR (NCBI non-redundant protein sequences), Swiss-Prot (A manually annotated and reviewed protein sequence database), and Pfam (protein family) for further analysis.

The Fragments Per Kilobase per Million mapped fragments (FPKM) value of each gene was calculated using Cufflinks (see text footnote 5), and the read counts of each gene were obtained by HTSeq package 2010^6^. Principal component analysis of the six RNA libraries was performed using the fast prcomp function (Molecular Devices, LLC, CA, United States), in which a score matrix was used to select probe sets that best fit the first principal component (PC1) and PC2. Differentially expressed genes (DEGs) were identified using the DEGSeq^7^, and *p*-adjust <0.001 and | log2FC| > = 1 were set as the significant differential expression threshold. Based on hypergeometric test and Bonferroni adjustment [corrected *p*-value (FDR) ≤0.05], KEGG pathway enrichment of the DEGs was analyzed. Short Time-series Expression Miner (STEM) was used to analyze and cluster expression trends of DEGs in response to SAHS stresses, clustered the DEGs into 50 expression profiles. Profiles with *p* < 0.01 were further separately subjected to the KEGG database for pathway enrichment, in which pathways with *p* < 0.01 were focused as significantly enriched pathways. All the above bioinformatics analysis was based on the integrated cloud platform of I-Sanger^8^, which was similar to that in our previous report (Hu et al., 2021). All sequence data from this study have been deposited in the public NCBI Sequence Read Archive (SRA) database under accession No. PRJNA675306.

### RNA Isolation and Real-Time Quantitative Reverse Transcriptase PCR

According to the manufacturer’s protocols, RNA of the *Haematococcus pluvialis* samples was isolated using an RNA fast 200 kit (Fastagen, Shanghai, China). Then, PrimeScript^TM^ RT reagent Kits with gDNA Eraser (Perfect Real Time) (TaKaRa, Japan) were used to perform the first-strand complementary DNA (cDNA) synthesis. cDNA was synthesized using oligo(dT) primers through reverse transcription with a total of 0.5 μg RNA in a 20-μl reaction system. A total of 16 genes, including eight kinds of carotenoid biosynthesis-related genes, seven fatty acid biosynthesis-related genes, and the β-actin gene, were selected for real-time fluorescence quantitative PCR (qRT-PCR) to investigate their expression profiles in response to SAHS treatment. The expression level of these genes was normalized to β-actin as the reference gene, as in previous studies (Eom et al., 2006; Lei et al., 2012; Ma et al., 2018; Hu et al., 2021). For qRT-PCR, gene-specific primers and methods were consistent with that reported in our previous study (Hu et al., 2021). The tested gene expression levels were determined using 2^–ΔΔCT^ values (Livak and Schmittgen, 2001).

### Statistical Analysis

The experiments were conducted with biological triplicate from separate microalgal cultures except for the transcriptome analysis. Data in the figures and tables were shown as the average of triplicate, and error bars were standard errors. One-way ANOVA (SPSS 19.0) was performed for statistical analysis, and *P*-values of less than 0.05 were considered as statistically significant.

## Results

### Microscopy and Growth Measurements

**FIGURE 1:**
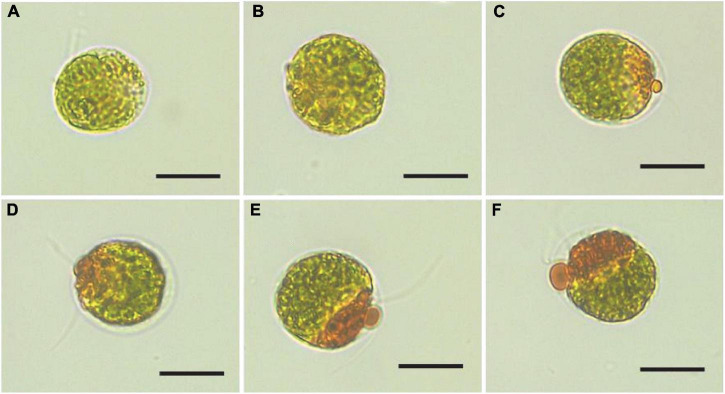
Microscopic images of *Haematococcus pluvialis* cells at different SAHS treatment stages. **(A–F)** represent the samples of Control, SAHS_1, SAHS_6, SAHS_12, SAHS_24, and SAHS_48, respectively. Scale bar = 20 μm.

In this study, visual observations revealed that color of the *Haematococcus pluvialis* cultures changed from green to lime green during the SAHS treatment from Stage Control to Stage SAHS_48. Morphological changes of *H. pluvialis* cells at the different SAHS treatment stages were also observed, displaying that the orange-red partial of the cells increased with treatment time extension (Control, SAHS_1, SAHS_6, SAHS_12, SAHS_24, and SAHS_48) (Figure 1), which might indicate that the content of carotenoid or astaxanthin in *H. pluvialis* cells increased during the 2-day incubation. Interestingly, flagella loss of the *H. pluvialis* cells was not observed in this study.

The growth pattern of *H. pluvialis* under SAHS treatment was also measured and showed in Table 1. All the treatments were started with the same cell density (CD) (9.92 ± 1.12 × 10^4^ cells⋅ml^–1^) and displayed a fluctuation level during the treatment stages, indicating that SAHS stresses did not cause cell death during the 2-day incubation. As revealed in Table 1, DCW from the treatments gradually increased with the treatment time. DCW at the early treatment stage, e.g., control, was significantly lower than the later stages. While DCW at later treatment stages, including SAHS_24 and SAHS_48, was significantly higher than the early stages, demonstrating that biomass growth was improved during the 2-day incubation. Carotenoid content (CC) accumulated in *H. pluvialis* cells remained the same in the early treatment stages until SAHS_6 and significantly increased during stage transitions with the treatment time extension. Single-cell carotenoid content (SCCC) displayed a similar change pattern with CC, which was at a low level at the early treatment stages and significantly increased during the later stages. The highest SCCC was 11.82 ± 0.44 pg⋅cell^–1^ achieved during Stage SAHS_48, about 135.46% higher than Stage Control. Astaxanthin content (AC) under SAHS treatment was 0.77 ± 0.04, 0.76 ± 0.04, 0.79 ± 0.03, 0.93 ± 0.02, 0.88 ± 0.08, and 1.16 ± 0.01 mg⋅L^–1^ in Control, SAHS_1, SAHS_6, SAHS_12, SAHS_24, and SAHS_48, respectively. AC in *H. pluvialis* cells also showed a similar change pattern with SCCC, and the highest AC at Stage SAHS_48 was about 50.65% compared with Stage Control. A basal level of total fatty acids (TFA) of 154.56 ± 16.56 mg⋅g^–1^ by DCW was measured in this study that increased significantly with the treatment time extension. The high TFAC at Stage SAHS_48 (210.10 ± 15.8 mg⋅g^–1^ by DCW) was about 35.96% higher than the lowest (154.56 ± 16.56 mg⋅g^–1^ by DCW) at Stage Control.

**TABLE 1:** Effects of SAHS treatments on growth measurements of *Haematococcus pluvialis.*

**Stages Measurements**	**Control**	**SAHS_1**	**SAHS_6**	**SAHS_12**	**SAHS_24**	**SAHS_48**
CD	9.92 ± 1.12^ab^	7.44 ± 0.86^c^	11.31 ± 0.39^*a*^	8.92 ± 0.24^bc^	10.58 ± 0.68^ab^	10.23 ± 0.67^ab^
DCW	0.13 ± 0.01^c^	0.16 ± 0.02^bc^	0.20 ± 0.02^ab^	0.20 ± 0.02^ab^	0.22 ± 0.02^a^	0.24 ± 0.02^a^
CC	0.49 ± 0.01^d^	0.43 ± 0.01^d^	0.43 ± 0.07^d^	0.67 ± 0.03^c^	0.96 ± 0.06^b^	1.21 ± 0.03^a^
SCCC	5.02 ± 0.54^d^	5.86 ± 0.67^d^	3.81 ± 0.76^e^	7.53 ± 0.40^c^	9.05 ± 0.43^b^	11.82 ± 0.44^a^
AC	0.77 ± 0.04^cd^	0.76 ± 0.04^d^	0.79 ± 0.03^cd^	0.93 ± 0.02^b^	0.88 ± 0.08^bc^	1.16 ± 0.01^a^
TFAC	154.56 ± 16.56^c^	165.30 ± 17.13^c^	162.06 ± 6.25^c^	186.74 ± 9.75^bc^	223.69 ± 18.80^a^	210.10 ± 15.8^ab^

*Data are given as means ± SD, *n* = 3. CD, cell density (× 10^4^ cell⋅ml^–1^); DCW, dry cell weight (mg⋅L^–1^); CC, carotenoids content (mg⋅L^–1^); SCCC, single cell carotenoids content (pg⋅cell^–1^); AC, astaxanthin content (mg⋅L^–1^); TFAC, total fatty acids content (mg⋅g^–1^); Lowercase letters indicate significant differences (*p* < 0.05) among the treatments.*

### Sequencing, *de novo* Transcriptome Assembly and Annotation

The molecular basis of the morphological and growth differences in *Haematococcus pluvialis* cells upon exposure to SAHS treatment was described based on a time course-dependent gene expression profiling, which was generated by RNA-Seq analyses performed on the alga samples. In total, six libraries were constructed and analyzed. The high throughput RNA sequencing obtained over 8.23 Gb clean data from each of the microalgal RNA libraries with Q20 all higher than 97.88% and a low-quality reads rate lower than 0.03% (Table 2). GC contents of the libraries ranged from 59.31 to 59.61%, and the mapping rate of all clean reads was higher than 91.46% (Table 2).

**TABLE 2:** RNA sequencing and mapping results.

**Algal RNA libraries**	**Raw reads**	**Clean reads**	**Clean bases (nt)**	**Error rate (%)**	**Q20 (%)**	**GC content (%)**	**Mapping rate (%)**
Control	51,078,628	50,323,266	7,453,145,620	0.02	98.03	59.41	93.87
SAHS_1	52,323,726	51,666,152	7,693,273,478	0.02	98.12	59.39	93.22
SAHS_6	49,328,446	48,739,818	7,254,036,353	0.02	98.15	59.61	93.59
SAHS_12	51,611,652	50,932,110	7,568,702,271	0.02	98.03	59.6	93.21
SAHS_24	51,642,478	50,784,936	7,538,385,545	0.03	97.88	58.98	91.88
SAHS_48	51,786,720	50,980,450	7,557,044,313	0.02	98.10	59.31	91.46

**FIGURE 2:**
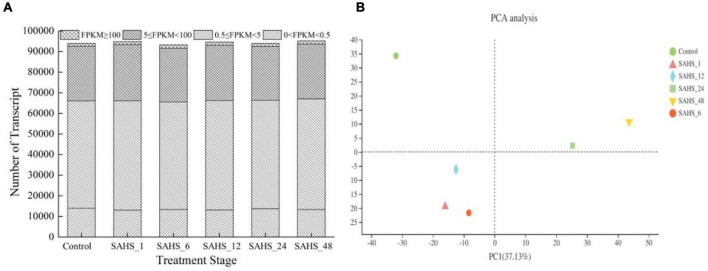
Global expression profiling of *H. pluvialis* unigenes at different treatment stages. **(A)** Numbers of detected unigenes in each library. **(B)** Principal component analysis of the RNA-Seq libraries.

Assembly of the reads generated a total of 112,886 unigenes, whose size varied from 69 to 38,640 bp with an average length of 1,409 bp and N50 of 1,959 bp, and a total length of 86,419,450 bp. Generally, 93,942, 94,780, 93,220, 94,517, 93,946, and 95,137 transcripts were found transiently expressed and induced by SAHS at stages of Control, SAHS_1, SAHS_6, SAHS_12, SAHS_24, and SAHS_48, respectively. The transcripts numbers identified in each *H. pluvialis* RNA library, expressed in FPKMs, are shown in Figure 2. In general, approximately 55% of the transcripts were in the 0.5–5 FPKM range, and 28% in the range of 5–100 FPKM (Figure 2A). Principal component analysis revealed that the six libraries could be assigned to separated groups (Figure 2B), suggesting that the overall transcriptome profiling of *H. pluvialis* was different at each SAHS treatment stage. Thus, the result distinguished *H. pluvialis* with responding to SAHS stresses at different stages. According to the PC analysis, the six libraries were assigned to three groups, in which stages SAHS_1, SAHS_6, and SAHS_12 were clustered together, suggesting that their overall transcriptome profiling was similar. Stages SAHS_24 and SAHS_48 were clustered together, and Stage Control clustered separately. Overall, a total of 112,886 unigenes and 61,323 genes were annotated by BLAST in the six databases. Specifically, 25,758 (40.28%), 25,100 (39.25%), 17,459 (27.30%), 33,350 (52.15%), 20,205 (31.60%), and 24,942 (39.00%) genes were annotated in COG, GO, KEGG, NR, Swiss-Prot, and Pfam, respectively (Table 3).

**TABLE 3:** The *de novo* transcriptome assembly and annotation results of unigenes in public protein databases.

**Statistics**	**Results**
**Assembly**	
Total unigenes	112,886
Total genes	61,323
Total length (bp)	86,419,450
Mean length (bp)	1,409
N50	1,959
**Annotation**	
Annotated in COG	25,758 (40.28%)
Annotated in GO	25,100 (39.25%)
Annotated in KEGG	17,459 (27.30%)
Annotated in NR	33,350 (52.15%)
Annotated in Swiss-Prot	20,205 (31.60%)
Annotated in PFAM	24,942 (39.00%)

### Differentially Expressed Genes

Cluster analysis of the differentially expressed genes (DEGs) was conducted to judge expression patterns of DEGs at different treatment stages. Pairwise comparisons were conducted among the six stages to identify DEGs correlating with SAHS, which revealed the expression changes of global genes during the 2-day incubation. As shown in Table 4, differential expression analysis revealed gene expression changes, and the numbers of upregulated or downregulated DEGs were displayed during the treatment stage transitions. For example, SAHS treatment stage transition from Control to SAHS_48 had the highest DEGs number (11,313) with 5,998 upregulated and 5,315 downregulated genes, while Stage SAHS_1 relative to SAHS_6 had the fewest DEGs (2,858), in which 2,051 genes were significantly downregulated, and 807 were upregulated. The stage transition from SAHS_24 to SAHS_48 also had a few DEGs numbers (3,350) with 2,374 upregulated and 976 downregulated genes. Stage Control relative to SAHS_1 had DEGs number of 6,411, in which 3,316 genes were significantly upregulated and 2,425 were downregulated. Stage transition of SAHS_6 to SAHS_12 had DEGs number of 3,822 with 2,565 significantly upregulated and 1,257 significantly downregulated genes. While stage SAHS_12 relative to SAHS_24 had a high DEGs number (6,941), in which 3,244 genes were significantly upregulated, and 3,697 were significantly downregulated.

**TABLE 4:** Numbers of DEGs involved in SAHS treatment stage transitions.

**Stages**	**CN**	**SAHS_1**	**SAHS_6**	**SAHS_12**	**SAHS_24**	**SAHS_48**
CN	–	3,985/2,425	3,316/4,011	3,395/2,052	5,281/5,145	5,998/5,315
SAHS_1	2,425/3,985	–	807/2,051	1,401/1,896	3,404/4,092	4,444/4,270
SAHS_6	4,011/3,316	2,051/807	–	2,565/1,257	3,047/1,538	4,665/2,322
SAHS_12	3,395/2,052	1,896/1,041	1,257/2,565	–	3,244/3,697	4,541/3,912
SAHS_24	5,145/5,281	4,092/3,404	1,538/3,047	3,697/3,244	–	2,374/976
SAHS_48	5,315/5,998	4,270/4,444	2,322/4,665	3,912/4,541	976/2,374	–

### Gene Expression Profiling and Kyoto Encyclopedia of Genes and Genomes Pathway Enrichments

**FIGURE 3:**
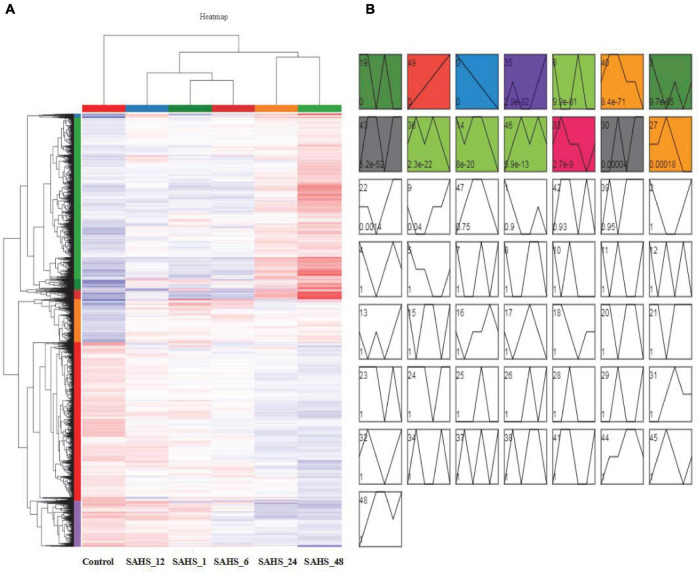
Cluster analysis and gene expression profiling of DEGs. **(A)** Cluster analysis of the DEGs. The *X* axis indicates sample type, the *Y* axis represents relative expression levels, and the red and blue represent upregulation and downregulation. **(B)** Gene expression profiling of DEGs by STEM analysis. The 14 colored profiles are significant profiles (*p* < 0.01), and the *P*-values in each cluster is shown.

Gene expression patterns throughout SAHS treatment were classified into 50 profiles, and STEM analysis demonstrated that 14 profiles, including Profiles 19, 49, 0, 35, 6, 40, 3, 43, 36, 14, 46, 33, 30, and 27, had *P*-values lower than 0.01 (Figure 3). The numbers of genes and significant enriched KEGG pathways of these profiles are shown in the Supplementary Table 1.

According to the results, the expression pattern of 2,062 genes in Profile 49 was upregulated during the treatment stages from Control to SAHS_48 in a row. The significantly enriched (*p* < 0.01) KEGG pathways were related to “fatty acid elongation,” “fatty acid biosynthesis,” “fatty acid degradation,” “prodigiosin biosynthesis,” “cutin, suberin, and wax biosynthesis,” “amino sugar and nucleotide sugar metabolism,” “fructose and mannose metabolism,” “ascorbate and aldarate metabolism,” “protein processing in the endoplasmic reticulum,” “MAPK signaling pathway plant,” “protein export,” and “plant hormone signal transduction.” And, in the profile, 35,954 genes were lowly expressed at early SAHS treatment stages, and highly expressed at Stage SAHS_48. The significantly enriched KEGG pathways were related to “fatty acid elongation,” “photosynthesis,” “cutin, suberin, and wax biosynthesis,” and “thermogenesis.” The 809 genes in Profile 46 were lowest at Stage Control, and then highly expressed in other stages. Two KEGG pathways were significantly enriched in this profile, which were “nitrogen metabolism” and “aminoacyl-tRNA biosynthesis.” The significantly enriched KEGG pathways in these profiles were similar, to a certain extent. These pathways were mostly related to secondary metabolite metabolism, cell wall synthesis, and signal transduction, while the 1,717 genes downregulated during the treatment stages from Control to SAHS_48 in a row were classified in Profile 0. For the genes in other significantly classified profiles, including Profiles 40, 3, 36, 14, and 27, they were highly expressed in early treatment stages, and lowly expressed in late treatment stages. The significantly enriched KEGG pathways in these profiles were composed of “citrate cycle (TCA cycle),” “fatty acid biosynthesis,” “phenylalanine, tyrosine, and tryptophan biosynthesis,” “pantothenate and CoA biosynthesis,” “other types of O-glycan biosynthesis,” “biosynthesis of unsaturated fatty acids,” “ribosome biogenesis in eukaryotes,” “parathyroid hormone synthesis, secretion, and action,” “tryptophan metabolism,” “beta-alanine metabolism,” “alanine, aspartate, and glutamate metabolism,” “purine metabolism,” “pyruvate metabolism,” “propanoate metabolism,” “glycine, serine, and threonine metabolism,” “thiamine metabolism,” “sphingolipid metabolism,” “synthesis and degradation of ketone bodies,” “valine, leucine, and isoleucine degradation,” “lysine degradation,” “ABC transporters,” “peroxisome,” “glycolysis/gluconeogenesis,” “the AGE-RAGE signaling pathway in diabetic complications,” “relaxin signaling pathway,” “MAPK-signaling pathway plant,” “phosphatidylinositol signaling system,” “human cytomegalovirus infection,” and “homologous recombination.” These pathways were mostly related to the regulation of stress response and cellular proliferation, apoptotic regulation, membrane transport, and hormone biosynthesis.

There were 976 genes in Profile 43; they showed low-expression levels at Stage Control and SAHS_12 and high-expression levels at the other stages. The significantly enriched KEGG pathways in this profile were “biosynthesis of terpenoids and steroids,” “selenocompound metabolism,” “carotenoid biosynthesis,” and “fatty acid elongation.” These pathways were mostly related to secondary metabolites biosynthesis.

According to the above results, genes that highly expressed at early SAHS treatment stages and lowly expressed at late stages were classified in Profiles 49, 35, and 46, and they were significantly enriched into pathways involved in fatty acid biosynthesis, secondary metabolism biosynthesis, signaling pathway, and so on. While the genes lowly expressed at early SAHS treatment stages and highly expressed at late stages were classified in Profiles 0, 40, 3, 36, 14, and 27, which were significantly enriched into KEGG pathways involved in regulation of stress response and cellular proliferation, apoptotic regulation, membrane transport, and hormone biosynthesis. The biosynthesis pathways of secondary metabolites we are interested in, for example, terpenoids and carotenoid, were also enriched in Profile 43. Under SAHS stresses, our study focused on carbon fixation, carotenoid biosynthesis, and fatty acid biosynthesis pathways.

### Effects of Salicylic Acid and High Concentration of Sodium Acetate Treatment on Specific Biological Pathways

In *Haematococcus pluvialis*, the maintenance of cellular homeostasis and biosynthesis of primary and secondary metabolites, e.g., fatty acids and astaxanthin, should generate a significant metabolic activity level and considerable demand for energy under stress conditions. Hence, our attention was focused on three specific pathways based on the analysis of DEGs in KEGG enrichment pathways, including “carbon fixation in photosynthetic organisms,” “fatty acid biosynthesis,” and “carotenoid biosynthesis” to explore the specific effects of the SAHS induction treatment stages (Control, SAHS_1, and SAHS_48) on *H. pluvialis*.

### Transcriptome and Pathway Analysis in Carbon Fixation

“Carbon fixation in photosynthetic organisms” in the KEGG database was generated to construct the carbon metabolism pathway of *H. pluvialis*. The results of Stage transition of SAHS_1 vs. Control and SAHS_48 vs. SAHS_1 revealed that key enzymes belong to the Calvin—Benson cycle (C_3_ pathway), C_4_-Dicarboxylic acid cycle (C_4_ pathway), and CAM pathway, e.g., phosphoenolpyruvate carboxylase (PEPC), pyruvate orthophosphate dikinase (PPDK), and malate dehydrogenase (MDH) were observed in *H. pluvialis*.

Further analysis determined the changes in carbon fixation pathways during Stage transition from Control to SAHS_1 and SAHS_1 to SAHS_48. During Stage transition of SAHS_1 vs. Control, four enzymes were upregulated, whereas two were downregulated in the C_4_ pathway (Table 5). Expression levels of the phosphoenolpyruvate carboxylase (PPC, EC: 4.1.1.31), aspartate transaminase (AST, EC: 2.6.1.1), and phosphoenolpyruvate carboxykinase (ATP) (PEPCK, EC: 4.1.1.49) increased by 16.30-, 2.13-, and 19.95-fold, respectively, in the C_4_ pathway. The expression level of malate dehydrogenase (oxaloacetate-decarboxylating) (NADP+) (MDH, EC: 1.1.1.40), which catalyzes the oxidative decarboxylation of (S)-malate in the presence of NADP^+^ and divalent metal ions, and decarboxylation of oxaloacetate, decreased by 2.33-fold. Conversely, one enzyme was upregulated, and eight downregulated in the C_3_ pathway, indicating that the key enzymes were downregulated. Expression levels of the downregulated genes decreased by 2.25- to 14.17-fold. Only the expression level of glyceraldehyde-3-phosphate dehydrogenase (GAPDH, EC: 1.2.1.12) increased in the C_3_ pathway by 5.30-fold. According to the results, when the culture environment shifted from control to SAHS treatment, the genes involved in the C_3_ pathway theoretically showed lower enzyme activity to help cells overcome the SAHS stresses. The downregulation of the expression levels of the genes might be due to high acetate concentrations. These enzymes created a counter-clockwise network, thereby reducing the content of 3-glycerol phosphate, starch, and sucrose in the cells. In addition, one enzyme was upregulated and one downregulated in the CAM pathways. For example, the expression level of the PEPC (EC: 4.1.1.31)-encoding gene, which is specific to CO_2_ fixation in algal cells, increased by 16.30-fold. However, the expression of MDH (EC: 1.1.1.40) decreased by 2.33-fold.

**TABLE 5:** The up and downregulated genes related to the carbon fixation pathways during SAHS Stage transitions.

**KEGG Orthology Category Description**	**SAHS_1 vs. Control**		**SAHS_48 vs. SAHS_1**	
	**Regulate mode**	**FC**	**Regulate mode**	**FC**
**C4-Dicarboxylic acid cycle (C_4_ pathway)**				
PEPC; Phosphoenolpyruvate carboxylase [EC: 4.1.1.31]	Up	16.30	Up	2.09
PEPCK; Phosphoenolpyruvate carboxykinase (ATP) [EC: 4.1.1.49]	Up	19.95	Down	7.44
AST; Aspartate transaminase [EC: 2.6.1.1]	Up	2.13	Down	1.93
MDH; Malate Dehydrogenase (NADP+) [EC: 1.1.1.82]	Down	2.32	–	
ALT; Alanine Transaminase [EC: 2.6.1.2]	–		Up	2.53
MDH; Malate Dehydrogenase (oxaloacetate-decarboxylating) (NADP+) [EC: 1.1.1.40]	Down	2.33	Down	2.62
**Calvin-Benson cycle (C_3_ pathway)**				
FBA; Fructose-bisphosphate aldolase [EC: 4.1.2.13]	Down	3.04	Up	2.02
FBPase; Fructose-bisphosphatase [3.1.3.11]	Up/down	–	Up	6.45
TK; Transketolase [EC: 2.2.1.1]	Down	2.25	–	
G3PDH; Glyceraldehyde-3-phosphate Dehydrogenase (NADP+) (phosphorylating) [EC: 1.2.1.13]	Down	14.17	Up	5.93
GAPDH; Glyceraldehyde-3-phosphate Dehydrogenase (phosphorylating) [EC: 1.2.1.12]	Up	5.30	Up/down	–
RPI; Ribulose-phosphate 3-epimerase [EC: 5.1.3.1]	Down	2.30	Down	2.07
PRK; Phosphoribulokinase [EC: 2.7.1.19]	Down	3.44	Up	2.49
PGK; Phosphoglycerate kinase [EC: 2.7.2.3]	Down	4.14	Up/down	–
Rubisco; Ribulose-bisphosphate carboxylase [EC: 4.1.1.39]	Up/down	–	Up	2.34
**CAM pathway**				
PEPC; Phosphoenolpyruvate carboxylase [EC: 4.1.1.31]	Up	16.30	Up	2.09
MDH; Malate Dehydrogenase (oxaloacetate-decarboxylating) (NADP+) [EC: 1.1.1.40]	Down	2.33	Down	2.62

*FC, fold change.*

During Stage transition from SAHS_48 to SAHS_1, four enzyme-encoding genes were upregulated and two downregulated in the C_3_ pathway. And there were two enzymes that were upregulated, and three were downregulated in the C_4_ pathway. Additionally, one enzyme was upregulated, and one downregulated in the CAM pathway. As estimated, the concentrations of the two upregulated enzymes, including PEPC and alanine transaminase (ALT, EC: 2.6.1.2), increased by 2.09 and 2.53 folds, respectively, in the C_4_ pathway. However, expression levels of three downregulated enzymes in this pathway, PEPCK, AST, and MDH, decreased by 7.44, 1.93, and 2.62-fold, respectively. The expression levels of fructose-bisphosphatase (FBPase, EC: 3.1.3.11) and glyceraldehyde-3-phosphate dehydrogenase (NADP+) (phosphorylating) (G3PDH, EC: 1.2.1.13) increased 6-fold in the C_3_ pathway, and the only downregulated enzyme in this pathway, ribulose-phosphate 3-epimerase (RPI, EC: 5.1.3.1), decreased by 2.07-fold. In the CAM pathways, PEPC expression increased by 2.09-fold, and MDH expression levels decreased by 2.62-fold during the stage transition from SAHS_1 to SAHS_48.

### Transcriptome and Pathway Analysis Involved in Carotenoid Biosynthesis

It was reported that the carotenoid biosynthesis-related genes were also upregulated with the increase in inorganic carbon (Wen et al., 2015). The universal isoprenoid precursor (IPP) and dimethylallyl pyrophosphate (DMAPP), the allylic isomer of IPP, were synthesized through the non-mevalonate pathway. The condensation reaction of IPP and DMAPP yields geranylgeranyl pyrophosphate (GGPP) for primary carotenoid biosynthesis (Lemoine and Schoefs, 2010).

According to the results, seven enzymes were upregulated, whereas three were downregulated in the carotenoid biosynthesis pathway during Stage transition of SAHS_1 vs. Control. Phytoene synthase (PSY, EC: 2.5.1.32) catalyzes the condensation of two GGPP molecules into one phytoene, which was 5.05-fold upregulated upon exposure to the SAHS stresses (Table 6). Phytoene is further converted to β-carotene by the successively catalyzing 15-*cis*-phytoene desaturase (PDS, EC: 1.3.5.5), 9,9′-*di*-*cis*-ζ-carotene desaturase (ZDS, EC: 1.3.5.6), and lycopene β-cyclase (CRTL-b, EC 5.5.1.19), which were upregulated by 3.09-, 3.77-, and 2.10-fold, respectively, under SAHS stresses. PDS is a rate-limiting enzyme of the carotenoid biosynthesis pathway, and the upregulation of its expression level may confer elevated astaxanthin biosynthesis capacity (Li et al., 2010). According to the results reported by Kobayashi (2003), astaxanthin is produced from β-carotene *via* two pathways, i.e., hydroxylation and oxidation of β-carotene catalyzed by β-carotene 3-hydroxylase (CrtZ, EC: 1.14.15.24) and β-carotene 4-ketolase (CrtW, EC: 1.14.99.63), which were upregulated by 3.65- and 4.24-fold, indicating enhanced astaxanthin production in *H. pluvialis* cells as a quick response to SAHS stresses.

**TABLE 6:** The up and downregulated genes related to the carotenoid biosynthesis pathway during SAHS stage transitions.

**KEGG Orthology Category Description**	**SAHS_1 vs. Control**		**SAHS_48 vs. SAHS_1**	
	**Regulate mode**	**FC**	**Regulate mode**	**FC**
PSY; 15-*cis-*phytoene synthase [EC: 2.5.1.32]	Up	5.05	Down	1.53
PDS; 15-*cis*-phytoene desaturase [EC: 1.3.5.5]	Up	3.09	Down	2.04
ZDS; 9,9′-*di*-*cis*-ζ-carotene desaturase [EC: 1.3.5.6]	Up	3.77	Down	4.17
CrtL-e; Lycopene ε-cyclase [EC: 5.5.1.18]	Down	7.67	Up	5.37
CrtL-b; Lycopene β-cyclase [EC: 5.5.1.19]	Up	2.10	–	
LUT5, CYP97A3; β-ring hydroxylase [EC: 1.14.-.-]	Down	2.52	–	
ZEP; zeaxanthin epoxidase [1.14.15.21]	Down	Inf	Up	Inf
CrtZ; β-carotene 3-hydroxylase [EC: 1.14.15.24]	Up	3.65	–	
CCD8; carlactone synthase [EC: 1.13.11.69]	Up	4.30	Up	3.08
CrtW; β-carotene 4-ketolase [1.14.99.63]	Up	4.24	–	

*FC, fold change; Inf, infinity.*

During Stage transition from SAHS_1 to SAHS_48, three kinds of enzyme-encoding genes involved in the carotenoid biosynthesis pathway were downregulated, including PSY, PDS, and ZDS, by 1.53-, 2.04-, and 4.17-fold (Table 6). The other three enzyme-encoding genes, including lycopene ε-cyclase (CrtL-e, EC: 5.5.1.18), zeaxanthin epoxidase (ZEP, 1.14.15.21), and carlactone synthase (CCD8, EC: 1.13.11.69), were upregulated in this pathway by 5.37, Inf, and 3.08-fold. These results indicated the downregulation of β-carotene and astaxanthin biosynthesis and enhanced accumulation of ε-carotene and violaxanthin during this Stage transition.

### Transcriptome and Pathway Analysis Involved in Fatty Acid Biosynthesis

It is well-known that astaxanthin is linearly correlated with the accumulation of fatty acids in *H. pluvialis* cells under environmental stresses, and over 96% astaxanthin is esterified by fatty acids to form astaxanthin ester and stored in triacylglycerol-rich cytoplasmic lipid droplets (Zhekisheva et al., 2002; Lei et al., 2012; Chen et al., 2015; Ma et al., 2018; Ding et al., 2019b; Pick et al., 2019; Cui J. et al., 2020). The increased production of neutral lipids is hypothesized to provide a solubility media for astaxanthin esters (Saha et al., 2013). Therefore, the formation of lipid droplets is a key factor driving and limiting astaxanthin accumulation in *H. pluvialis* (Cheng et al., 2017). As a result, any potential discrepancy of FAs upon exposure to SAHS stresses will be an important complement to the efficient astaxanthin accumulation knowledge of *H. pluvialis*.

During Stage transition from Control to SAHS_1, six enzyme-encoding genes involved in the fatty acid biosynthesis pathway were upregulated (Table 7). Acetyl-CoA carboxylase (ACC; EC: 6.4.1.2) and fatty acid synthase (FASN; EC: 2.3.1.85) begin the *de novo* fatty acid synthesis in eukaryotic algae (Harwood, 2019; Li-Beisson et al., 2019). ACC catalyzed the carboxylation of acetyl-CoA to generate malonyl-CoA (Hasan et al., 2018), which is the first committed and the rate-limiting step of fatty acid biosynthesis (Chen et al., 2019), and was upregulated by 2.45-fold. The sole enzyme, FASN, which is capable of the reductive *de novo* biosynthesis of long-chain fatty acids from nicotinamide adenine dinucleotide phosphate- NADPH-, malonyl-CoA, and acetyl-CoA (Ruth and Javier, 2006), was upregulated by 2.64-fold. Then, the enzyme [acyl-carrier-protein] *S-*malonyltransferase (FabD; EC: 2.3.1.39), which enhanced the production of malonyl-ACP from malonyl-CoA, was upregulated by 3.41-fold. Then, the acyl chain is elongated with the condensation reaction between malonyl-ACP and acyl-ACP in each turn until hexadecanoyl-ACP is finally formed (Sánchez and Harwood, 2002). Stearoyl-[acyl-carrier-protein] 9-desaturase (AAD; EC: 1.14.19.2) catalyzes the introduction of a double bond to the acyl group and esterifies to produce acyl-ACP (Rismani-Yazdi et al., 2011). The termination of the elongation of fatty acids is catalyzed by Oleoyl-[acyl-carrier-protein] hydrolase (OAH; EC: 3.1.2.14) *via* removing of an acyl group from acyl-ACP (Rismani-Yazdi et al., 2011). Long-chain-fatty-acid—CoA ligase (ACSL; EC: 6.2.1.3) activates the hexadecanoic acid through the esterification of hexadecanoyl-CoA and is further used in glycerolipid metabolism, glycerophospholipid metabolism, and fatty acid elongation. AAD, OAH, and ACSL were upregulated by 6.54-, 3.09-, and 2.85-fold, respectively. During Stage transition of SAHS_48 vs. SAHS_1, two kinds of enzymes were upregulated in the fatty acid biosynthesis pathway, including FASN and FabD, by 13.50- and 5.00-fold (Table 7). In addition, three enzymes (FabF, AAD, and OAH) were downregulated in this pathway by 2.75-, 3.35-, and 3.23-fold, respectively.

**TABLE 7:** The up and downregulated genes related to the fatty acid biosynthesis pathway during SAHS Stage transitions.

**KEGG Orthology Category Description**	**SAHS_1 vs. Control**		**SAHS_48 vs. SAHS_1**	
	**Regulate mode**	**FC**	**Regulate mode**	**FC**
FASN; Fatty-acid synthase [EC: 2.3.1.85]	Up	2.64	Up	13.50
ACC; Acetyl-CoA carboxylase [6.4.1.2]	Up	2.45	Down/up	–
FabD; ACP *S* -malonyltransferase [EC: 2.3.1.39]	Up	3.41	Up	5.00
FabF; β-ketoacyl-ACP synthase II [EC: 2.3.1.179]	–		Down	2.75
AAD; Stearoyl-ACP 9-desaturase [EC: 1.14.19.2]	Up	6.54	Down	3.35
OAH; Oleoyl-ACP hydrolase [EC: 3.1.2.14]	Up	3.09	Down	3.23
Long-chain-fatty-acid—CoA ligase [ACSL; EC: 6.2.1.3]	Up	2.85	Down/up	–

*FC, fold change.*

### Analysis of the Expression Levels by Real-Time Quantitative PCR

**FIGURE 4:**
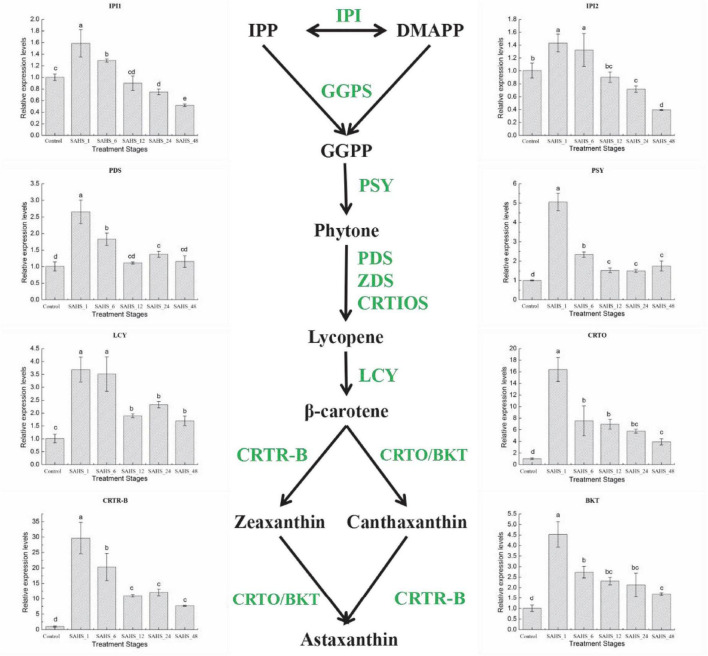
Expression of genes in the *H. pluvialis* astaxanthin biosynthesis pathway under different treatment stages. Lower case letters indicate significant differences (*p* < 0.05) among the treatments.

**FIGURE 5:**
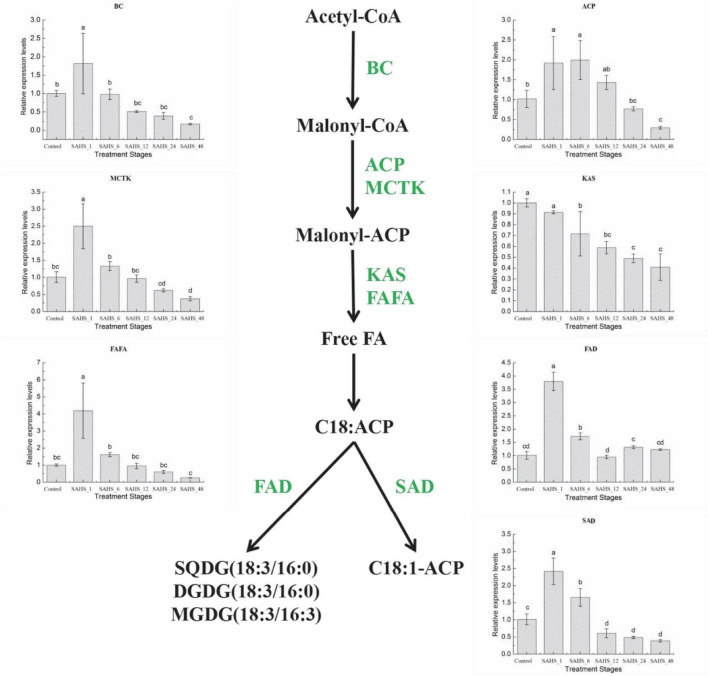
Expression of genes in the *H. pluvialis* fatty acids biosynthesis pathway under different treatment stages. Lower case letters indicate significant differences (*p* < 0.05) among the treatments.

In this study, expression levels of the key genes involved in carotenogenic and fatty acid biosynthesis pathways were determined during the SAHS treatment stages. The results demonstrated that expression levels of the studied genes varied among the stages (Figures 4, 5). To study the relationship between the genes involved in carotenogenic and fatty acid biosynthesis pathways, the Pearson’s correlation analysis (SPSS 19.0) was carried out, and the results are summarized in Table 8.

**TABLE 8:** Correlations between expression of carotenogenic and fatty acid biosynthesis genes (cofactors, Pearson correlation in SPSS 19).

**Genes**	**IPI1**	**IPI2**	**PDS**	**PSY**	**LCY**	**CRTO**	**CRTR_B**	**BKT**	**BC**	**ACP**	**MCTK**	**KAS**	**FAFA**	**FAD**	**SAD**
IPI1	1	0.927**	0.796**	0.769**	0.692**	0.684**	0.700**	0.704**	0.911**	0.755**	0.850**	0.657**	0.896**	0.784**	0.926**
IPI2	0.927**	1	0.705**	0.618**	0.699**	0.602**	0.664**	0.661**	0.792**	0.798**	0.844**	0.741**	0.732**	0.633**	0.874**
PDS	0.796**	0.705**	1	0.934**	0.853**	0.913**	0.932**	0.910**	0.734**	0.647**	0.873**	0.304	0.824**	0.934**	0.884**
PSY	0.769**	0.618**	0.934**	1	0.771**	0.915**	0.872**	0.909**	0.749**	0.503*	0.825**	0.317	0.903**	0.983**	0.845**
LCY	0.692**	0.699**	0.853**	0.771**	1	0.799**	0.925**	0.861**	0.530*	0.616**	0.691**	0.181	0.652**	0.739**	0.754**
CRTO	0.684**	0.602**	0.913**	0.915**	0.799**	1	0.952**	0.967**	0.597**	0.611**	0.836**	0.189	0.770**	0.881**	0.760**
CRTR_B	0.700**	0.664**	0.932**	0.872**	0.925**	0.952**	1	0.951**	0.556*	0.669**	0.806**	0.172	0.709**	0.841**	0.790**
BKT	0.704**	0.661**	0.910**	0.909**	0.861**	0.967**	0.951**	1	0.606**	0.639**	0.841**	0.234	0.767**	0.874**	0.774**
BC	0.911**	0.792**	0.734**	0.749**	0.530*	0.597**	0.556*	0.606**	1	0.535*	0.779**	0.678**	0.930**	0.792**	0.881**
ACP	0.755**	0.798**	0.647**	0.503*	0.616**	0.611**	0.669**	0.639**	0.535*	1	0.788**	0.424	0.514*	0.483*	0.708**
MCTK	0.850**	0.844**	0.873**	0.825**	0.691**	0.836**	0.806**	0.841**	0.779**	0.788**	1	0.629**	0.798**	0.828**	0.913**
KAS	0.657**	0.741**	0.304	0.317	0.181	0.189	0.172	0.234	0.678**	0.424	0.629**	1	0.531*	0.389	0.644**
FAFA	0.896**	0.732**	0.824**	0.903**	0.652**	0.770**	0.709**	0.767**	0.930**	0.514*	0.798**	0.531*	1	0.916**	0.878**
FAD	0.784**	0.633**	0.934**	0.983**	0.739**	0.881**	0.841**	0.874**	0.792**	0.483*	0.828**	0.389	0.916**	1	0.856**
SAD	0.926**	0.874**	0.884**	0.845**	0.754**	0.760**	0.790**	0.774**	0.881**	0.708**	0.913**	0.644**	0.878**	0.856**	1

*Indicated that there was a significant correlation between the expression genes at the *p* < 0.05 level; **Indicated that there was a significant correlation between the expression genes at the *p* < 0.01 level.

### Transcript Expression of Carotenogenic Genes

Isopentenyl pyrophosphate isomerase (IPI) catalyzes the isomerization of IPP to DMAPP, and DMAPP is the precursor for the formation of the carotenoid backbone (Lee and Schmidt-Dannert, 2002). PDS catalyzes the first two dehydrogenation reactions to convert the pale yellow phytoene into ζ-carotene and phytofluene, which is one of the rate-limiting steps for carotenoid biosynthesis (Steinbrenner and Sandmann, 2006; Liu et al., 2013, 2014; Galarza et al., 2018). Then, phytoene, the first tetraterpene carotenoid in carotenoid biosynthesis pathway of *H. pluvialis*, is formed by PSY, a synthase catalyzing the head-to-tail condensation of two GGPP molecules (Gong and Bassi, 2016). The synthesis of phytoene is also a rate-limiting step for astaxanthin accumulation in *H. pluvialis* (Gong and Bassi, 2016). The flux of β-carotene from lycopene is catalyzed by lycopene β-cyclase (LCY; Gao et al., 2012a, b, 2013a; Galarza et al., 2018). Carotenoid oxygenase (CRTO) and carotenoid hydroxylase (BKT2) are two types of β-carotene ketolases/oxygenases that catalyze β-carotene to produce astaxanthin together with β-carotene hydroxylase (CRTR-B; Meng et al., 2005, 2006; Gao et al., 2010).

The expression levels of the eight carotenogenic genes varied among the stages (Figure 4). In this study, all of these genes were overexpressed at the transcript level during the 2-day cultivation. IPI1 and IPI2 had a similar expression pattern, both were over-expressed at a transcriptional level on Stage SAHS_1 and then significantly downregulated (*p* < 0.05) with treatment time extension. SAHS_48 showed the lowest expression level, which was significantly downregulated in comparison with the control. The highest expression levels of these genes were all achieved in Stage SAHS_1, which were significantly upregulated by 2.65 ± 0.35-, 5.06 ± 0.45-, 3.68 ± 0.48-, 16.41 ± 2.06-, 29.68 ± 5.06-, and 4.53 ± 0.60-fold in comparison with the Stage Control in PDS, PSY, LCY, CRTO, CRTR-B, and BKT, respectively. Moreover, expression levels of these genes were significantly downregulated along with the treatment time. According to the above results, the expression of the carotenogenic genes displayed a similar pattern. In general, expression of these genes was significantly upregulated after SAHS treatment for 1 h and then downregulated with treatment time extended. The expression levels of PDS, PSY, LCY, CRTO, CRTR-B, and BKT during all treatment stages were higher than that of control, resulting in continuous biosynthesis of carotene and astaxanthin. Posttranscriptional regulation of these genes was thought to play a role in astaxanthin accumulation.

### Transcript Expression of Fatty Acid Biosynthesis Genes

Biotin carboxylase is a subunit of acetyl coenzyme A carboxylase-biotin carboxylase, which catalyzes the carboxylation of acetyl-CoA to form malonyl-CoA, and is the first rate-limiting step of fatty acid biosynthesis in *H. pluvialis*. Acyl carrier protein (ACP) catalyzes a growing chain bound to a thiol ester at the distal thiol of a 4′-phosphopantetheine moiety, which acts as a vital component in both polyketide and fatty acid biosynthesis processes. Malonyl-CoA: ACP transacylase (MCTK) catalyzes the transfer of malonyl moiety from malonyl-CoA onto ACP, which is the fatty acid elongation step through extending the growing the length of acyl chain by two carbons. Furthermore, the condensation reaction between acetyl CoA and malonyl ACP is catalyzed by 3-ketoacyl-ACP synthase (KAS). In the fatty acid biosynthesis pathway, the chain-length-determining enzyme, Acyl-ACP thioesterase (FATA), catalyzes the hydrolysis of the thioester bond of acyl-ACP, releasing ACP and free fatty acid. Stearoyl-ACP-desaturase (SAD) plays an essential role in determining the ratio of saturated to unsaturated fatty acids by catalyzing the conversion of 18:0 to C18:1n9. And the conversion of C18:3n3 from C18:2n6 is catalyzed by ω-3 fatty acid desaturase (FAD; Zhao N. et al., 2015; Ma et al., 2018).

As shown in Figure 5, mRNA expression of most fatty acid biosynthesis genes, except for KAS, was significantly upregulated after SAHS treatment for 1 h and then downregulated according to the time extension. The highest expression level of these six genes was achieved during Stage SAHS_1, which were upregulated by 1.82 ± 0.82-, 1.92 ± 0.67-, 2.50 ± 0.66-, 4.19 ± 1.62-, 3.80 ± 0.35-, and 2.41 ± 0.39-fold in comparison with that of Stage Control in BC, ACP, MCTK, FAFA, FAD, and SAD, respectively. KAS was downregulated with the extension of SAHS treatment time in the 2-day incubation period. The lowest expression level for all these genes was achieved in Stage_48, downregulated by 0.17 ± 0.02-, 0.29 ± 0.03-, 0.37 ± 0.06-, 0.41 ± 0.12-, and 0.39 ± 0.03-fold in comparison with the Stage Controls BC, ACP, MCTK, KAS, FAFA, and SAD, respectively.

### Correlations Between the Expression of Carotenogenic and Fatty Acid Biosynthesis Genes

Pearson’s correlation analysis (SPSS 19.0) was carried out to study the relationship of gene expressions between the key genes involved in two important biosynthesis pathways, including carotenogenic and fatty acid, in *H. pluvialis*, and the results are summarized in Table 8.

According to the results in Table 8, the correlations between these genes were different, and no gene expression demonstrated a negative correlation with the others in this study. Expression levels of almost all these genes showed a significant or very significant correlation with that of the other genes, except KAS. The expression of KAS shared very significant correlations with IPI1 and IPI2 genes involved in the carotenogenic biosynthesis pathway and BC, MCTK, and SAD genes involved in the fatty acid biosynthesis pathway. The expression of KAS displayed a significant correlation with FAFA.

## Discussion

In this study, a time course-dependent gene expression profiling and carotenoid and fatty acid biosynthesis under SAHS stresses were studied. Astaxanthin accumulated in *H. pluvialis* cells is known to be deposited in cytoplasm lipid droplets (Pick et al., 2019). According to the growth pattern of this microalga, cell growth was improved during the incubation period, and the induced accumulation of carotenoid/astaxanthin and fatty acids in the single cells under SAHS treatment corresponded with the increased orange-red partial of the microalga cells (Figure 1 and Table 1). The results displayed that SAHS stresses not only enhanced carotenoid/astaxanthin and fatty acids production in *H. pluvialis* but also maintained its biomass growth, which might provide foundation to optimize the astaxanthin production efficiency in large scale *H. pluvialis* cultivation with commercial applications. Meanwhile, the accumulation of fatty acids during incubation indicated that SAHS stresses might be a suitable treatment condition for biodiesel production in *H. pluvialis*.

High-quality sequencing and assembly data were obtained for exploration of molecular processes or pathways involved in adaptation and pressure response of *H. pluvialis* to SAHS stresses in this study (Tables 2, 3). The results provided a comprehensive achievement of all functionally characterized genes involved in SAHS-responding determination, and several genes involved in carbon metabolism, primary and secondary metabolism, and immune system responses were identified. It was displayed that the numbers of DEGs decreased along with the sequential transition of the SAHS treatment stages. More DEGs were upregulated for most of the Stage transitions, suggesting that there were greater differences in global gene expression and functions in *H. pluvialis* during earlier response stages (Table 4). Higher transcriptional activity was observed during the immediate response of *H. pluvialis* to SAHS stresses, and its adaption to the stress conditions resulted in lower transcriptional activity. This result was similar to the PC analysis, indicating that the six libraries were assigned to three separated groups. STEM analysis displayed the 14 significant gene expression profiles under SAHS stresses. It is conceivable that a significant level of metabolic activity and considerable demand for energy should be generated to maintain the cellular homeostasis and biosynthesis of astaxanthin and lipids in *H. pluvialis* cells in response to stressful conditions.

### How Salicylic Acid and High Concentration of Sodium Acetate Treatment Affected the Specific Biological Pathways?

Analysis of the specific biological pathways showed that the main metabolic pathway in *H. pluvialis* under the SAHS stresses is the C_3_ pathway. *H. pluvialis* cells were induced to employ the CO_2_-concentrating mechanism (CCM) to adapt to the altered environmental conditions. After 1 h of SAHS stresses exposure, enzymes of the C_4_ pathway were upregulated immediately to produce CO_2_, which finally entered the C_3_ pathway. Under prolonged treatment, enzymes of the C_4_ pathway were upregulated to decompose malic acid into pyruvate, whereas pyruvate was an intermediate metabolite with important functions, taking part in the metabolism of three major nutrients (i.e., proteins, carbohydrates, and lipids). The enzymes of the C_3_ pathway were also upregulated during Stage transition from SAHS_1 to SAHS_48, indicating higher process efficiency of the C_3_ cycle. The enzymes in the CAM pathway displayed the same change pattern, which was upregulated to malic acid and then to pyruvate *via* decomposition. SA was thought to play a role in the adaption of *H. pluvialis* cells to HS stress, leading more carbon to flux into the synthesis of pigments, which can offer more help for the cells to adapt to low light conditions under the consistent HS stress. The biosynthesis of fatty acids in *H. pluvialis* cells was upregulated in response to SAHS stresses throughout the cultivation period. However, the biosynthesis of unsaturated fatty acids and long-chain fatty acids was upregulated initially and inhibited with time extension.

It was previously reported that intensive production of carbohydrates is the first response of *H. pluvialis* to abiotic stresses, which were then partially degraded to support fatty acid synthesis (Recht et al., 2012; Cheng et al., 2016; Li et al., 2017). In this study, the increased production of CO_2_ and pyruvate in the carbon fixation pathway enhanced the production of proteins, carbohydrates, and lipids. The increased expression of genes related to the carbon fixation pathway indicated that both SA and NaAC improved energy production of the microalgal cells, which means enhanced carbon assimilation and cell division (Sun et al., 2018; Fu et al., 2021), and meanwhile, increased production of astaxanthin and fatty acids in this study. The upregulation of genes involved in the carotenoid biosynthesis pathway enhanced the biosynthesis of astaxanthin and associated pigments. The upregulation of the expression levels of FASN, ACC, and FabD genes enhanced fatty acid biosynthesis, and, thus, promoted both the astaxanthin esterification and deposition pathways in *H. pluvialis* cells. SA was reported to downregulate the responsible genes for fatty acids biosynthesis, except for the SA24 group by RNA-Seq analysis (Gao et al., 2016). In this study, majority of the genes related to fatty acid biosynthesis in *H. pluvialis* were upregulated during the 1st-h SAHS treatment, and the genes involved in free fatty acid biosynthesis were upregulated during the 48-h cultivation. In contrast, the genes involved in unsaturated fatty acid biosynthesis were downregulated at 48 h in comparison with the SAHS_1 stage. Therefore, the induced fatty acid production during 48-h SAHS treatment was likely to provide astaxanthin storage capacity within *H. pluvialis* cells. Fatty acid biosynthesis was induced immediately after addition of high concentration NaAC in the *H. pluvialis* culture medium, which might be owing to NaAC provided both energy and carbon skeleton for the biosynthesis of fatty acid. The downregulation of unsaturated fatty acid biosynthesis genes in Stage SAHS_48 in comparison with Stage SAHS_1 was speculated to be caused by exogenous SA, which provided the protection mechanism of the microalga cells against the abiotic stress caused by high NaAC, for the unsaturated fatty acid could prevent *H. pluvialis* cells from damage by a reactive oxygen species.

In conclusion, our study speculated that SA and excessive NaAC provide both energy and precursors for astaxanthin and fatty acid biosynthesis with an effective mechanism in *H. pluvialis*. SA might act as a signaling molecule to improve cell growth of *H. pluvialis* and promote and drive the above physiological properties in the cells to provide self-protection metabolism for this microalga at the same time. This may be a matter of gene expression, and further attention is required to draw the conclusion.

### How Salicylic Acid and High Concentration of Sodium Acetate Treatment Affected the Expression Levels of Carotenoid and Fatty Acid Biosynthesis Genes?

Gao et al. (2015) revealed that the expression pattern of the five genes involved in the carotenoid biosynthesis pathway in *H. pluvialis*, including PDS, ZDS, CRTZ, CRTB, and ZEP, differed between salicylic acid and jasmonic acid inductions. Expression levels of the PSY, PDS, ZDS, and CRTR-B genes were upregulated, correlating with excessive accumulation of astaxanthin in *H. pluvialis* cells. They showed variable upregulated patterns under the stress of the same level of jasmonic acid or SA inductions. The downregulation of PSY, PDS, and LCYB during the first 2 days was considered to be caused by environmental changes and may exhibit certain synergy among the expression of these carotenogenic genes. They also demonstrated in another study that the transcriptional expression levels of eight carotenoid genes in *H. pluvialis* were all downregulated in the first 12 h (except BKT and PDS genes), follow SA25 treatment, and were less than 2.5-fold in comparison with Control follow SA25 treatment in 2-day cultivation (except PDS gene) (Gao et al., 2012b). The phytoene and astaxanthin biosynthesis-related genes were studied at the transcript level under NaAC stress by Cong et al. (2020), who reported that CrtW and CrtZ, the two key enzymes involved in astaxanthin biosynthesis, were continuously upregulated since the 1st day of induction, while expression levels of PSY, PDS, and LCYB genes were continuously upregulated ever since the 3rd day of induction. He et al. (2018) conducted systemic transcriptome analysis about three variables, including light, acetate, and Fe^2+^, to explore the molecular mechanism of astaxanthin accumulation in the red-cell stage of *H. pluvialis*. It was demonstrated that the expression of CRTZ gene was significantly promoted by acetate induction; acetate was further revealed to promote astaxanthin biosynthesis in *H. pluvialis* by enhancing the expression of CRTZ gene, and meanwhile, inhibiting the expression of LCYE gene. Based on the mode of β-actin gene of *H. pluvialis* under nitrogen starvation, Zhao et al. (2020) revealed that expression of the astaxanthin biosynthesis-related genes was significantly promoted to distribute carbon flow through the MEP pathway to the astaxanthin biosynthesis pathway permanently to defend the nitrogen starvation stress. In particular, expression levels of the most astaxanthin biosynthesis-related genes, including IPI, PSY, ZDS, CHYB, and BKT, all increased by more than 5-fold.

With regard to lipids, Gao et al. (2016) displayed that transcriptional expression of the fatty acid biosynthesis genes, including BC, MCTK, ACP, KAS, FAFA, FAD, and SAD, was in line with the RNA-Seq transcriptomic analysis. Expression levels of the seven genes were all decreased after SA treatment for 6 h, and the transcriptional levels of SA1 were similar to that of SA24, except for FAD gene, which was significantly higher in SA24 than SA1. It was found that the synthesis of storage lipid was stimulated substantially by high irradiance induction conditions and regulated by expression of *de novo* genes involved in the fatty acid biosynthesis pathway (Gwak et al., 2014). He et al. (2018) demonstrated that the genes involved in fatty acid-related pathways, especially the fatty acid elongation pathway, were significantly affected by acetate induction conditions and acetate-promoted expression of the lipid pathway-related genes in *H. pluvialis*. Addition of acetate in cultivation system of *H. pluvialis* further enhanced expression levels of the KCS and MECR genes. Zhao et al. (2020) revealed that all the transcripts associated with the *de novo* biosynthesis of fatty acids showed a higher expression level with nitrogen starvation.

In this study, the q-RT-PCR results indicated that most of the genes involved in our interested pathways revealed a similar expression pattern, which was upregulated immediately from Stage control to SAHS_1 and then decreased with the treatment time extension. Meanwhile, expression of the KAS gene showed a consistently decreasing trend with the time extension. However, expression of the IPI genes achieved the highest levels at Stage SAHS_48, while expression of PDS, PSY, LCY, CRTO, CRTR-B, and BKT genes displayed a similar or a higher level at Stage SAHS_48 compared with the control. The majority of the genes involved in the fatty acid biosynthesis pathway, including BC, ACP, MCTK, FAFA, FAD, and SAD, displayed the same expression pattern, which was significantly upregulated after SAHS treatment for 1 h and then downregulated with the treatment time extension (except KAS), and the transcriptional levels of Stage SAHS_48 were significantly lower than that of Stage Control, except for FAD, which was similar to Control. The above results were consistent with the RNA-Seq transcriptomic analysis, which revealed that addition of high concentration of NaAC immediately induced fatty acid and astaxanthin biosynthesis in *H. pluvialis* cells. And the upregulation of carotenoid biosynthesis genes resulted in continuous accumulation of astaxanthin in the microalga cells, which was observed. The downregulation of fatty acid biosynthesis-related genes, especially the unsaturated fatty acid biosynthesis genes, from Stage SAHS_1 to SAHS_48 demonstrated that exogenous SA played a role in providing a protection mechanism for *H. pluvialis* cells against the abiotic stress caused by high NaAC. These results were consistent with the astaxanthin, and fatty acid accumulation speculated to stimulate the self-protective metabolism as a defense against the high concentration sodium acetate stress (Cong et al., 2020).

The correlations between these genes were all positively correlated. The gene clusters involved in either carotenoid or fatty acid biosynthesis pathways significantly positively correlated, which might indicate that these two pathways were stoichiometrically coordinated at the transcript level. Our detailed correlation analysis between genes involved in these two pathways provided some interesting hints for the molecular mechanism involved in coordination between astaxanthin and fatty acid biosynthesis (Table 8). The correlation analysis between gene expressions in the two pathways might detect some new vital genes and provide insight into the coordination between them. SA was thought to act as a signaling molecule and play a role in inhibiting the upregulation of the fatty acid and astaxanthin biosynthesis genes through self-protection metabolism in *H. pluvialis*, leading to its adaption to HS stress and finally avoid the massive cell death, which was caused by excessive NaAC condition. These results were consistent with the previous study reported by Ma et al. (2018), which revealed that nitrogen starvation-induced gene expressions of astaxanthin and fatty acid-related genes (except for IPI1 and IPI2) corresponded to the accumulation of total astaxanthin and fatty acid content in *H. pluvialis*. It was deduced in this study that SA and NaAC provide both energy and precursors for astaxanthin and fatty acid biosynthesis with an effective mechanism in *H. pluvialis*. SA played a role in improving cell growth of *H. pluvialis*, promoting and driving physiological properties to provide self-protection metabolism in the microalga cells against the abiotic stress caused by excessive NaAC.

Hence, the results of this study provided the foundation to optimize the induction conditions leading to improving astaxanthin production efficiency in large-scale cultivation. Further studies on the molecular mechanism for lipid and astaxanthin accumulation *via* salicylic acid combined with sodium acetate are required.

## Data Availability Statement

The datasets presented in this study can be found in online repositories. The names of the repository/repositories and accession number(s) can be found below: https://www.ncbi.nlm.nih.gov/, PRJNA675306.

## Author Contributions

QH performed the experiments, analyzed the data, supervised the experimental work, and wrote the manuscript. MS performed the additional experiments. CW designed the study and formulated the manuscript. DH, ZH, and YW provided additional supervision and contributed to the final manuscript. All authors contributed to the article and approved the submitted version.

## Conflict of Interest

The authors declare that the research was conducted in the absence of any commercial or financial relationships that could be construed as a potential conflict of interest.

## Publisher’s Note

All claims expressed in this article are solely those of the authors and do not necessarily represent those of their affiliated organizations, or those of the publisher, the editors and the reviewers. Any product that may be evaluated in this article, or claim that may be made by its manufacturer, is not guaranteed or endorsed by the publisher.

## Footnotes

1
http://hannonlab.cshl.edu/fastx_toolkit

2
https://github.com/jstjohn/SeqPrep

3
https://github.com/najoshi/sickle

4
http://tophat.cbcb.umd.edu/

5
http://cole-trapnelllab.github.io/cufflinks/

6
http://www-huber.embl.de/users/anders/HTSeq

7
http://bioconductor.org/packages/stats/bioc/DEGSeq/

8
https://www.i-sanger.com/
