# Supplementary material for: Haematococcus pluvialis Accumulated Lipid and Astaxanthin in a Moderate and Sustainable Way by the Self-Protection Mechanism of Salicylic Acid Under Sodium Acetate Stress
Source: Front Plant Sci. 2021 Nov 19;12:763742. doi: 10.3389/fpls.2021.763742 (PMC8639525; doi:10.3389/fpls.2021.763742)
Supplement: Supplementary file 1 [file Table_1.DOCX]

**SUPPORTING INFORMATION**

TABLE S1 | The 14 significantly expressed profiles and their significantly enriched KEGG functional pathways.

| Profile | Number of genes | Description | Cluster Frequency (%) | P-value |
| --- | --- | --- | --- | --- |
| Profile 0 | 1717 | AGE-RAGE signaling pathway in diabetic complications | 1.65 | 5.84E-03 |
|  |  | Other types of O-glycan biosynthesis | 1.65 | 9.76E-03 |
|  |  | Relaxin signaling pathway | 1.89 | 5.31E-03 |
| Profile 3 | 1029 | Valine, leucine and isoleucine degradation | 3.66 | 5.43E-06 |
|  |  | MAPK signaling pathway - plant | 3.25 | 1.49E-03 |
|  |  | Phosphatidylinositol signaling system | 3.25 | 1.42E-03 |
|  |  | Human cytomegalovirus infection | 2.44 | 5.09E-03 |
| Profile 6 | 916 | Cellular senescence | 3.15 | 6.76E-03 |
|  |  | Kaposi sarcoma-associated herpesvirus infection | 2.36 | 6.45E-03 |
| Profile 14 | 910 | Parathyroid hormone synthesis, secretion and action | 2.51 | 5.34E-03 |
| Profile 19 | 2173 | Phagosome | 3.98 | 5.13E-07 |
| Profile 27 | 591 | Ribosome biogenesis in eukaryotes | 7.05 | 2.17E-05 |
| Profile 33 | 1230 | Citrate cycle (TCA cycle) | 2.73 | 1.46E-03 |
|  |  | Phenylalanine metabolism | 1.52 | 4.73E-03 |
|  |  | Isoquinoline alkaloid biosynthesis | 1.21 | 3.72E-03 |
| Profile 35 | 954 | Fatty acid elongation | 3.73 | 3.87E-06 |
|  |  | Photosynthesis | 4.97 | 4.29E-04 |
|  |  | Cutin, suberine and wax biosynthesis | 1.24 | 3.62E-03 |
|  |  | Thermogenesis | 6.21 | 7.99E-03 |
| Profile 36 | 870 | Purine metabolism | 8.50 | 4.63E-04 |
|  |  | Homologous recombination | 2.83 | 3.11E-03 |
|  |  | Parathyroid hormone synthesis, secretion and action | 2.83 | 2.75E-03 |
|  |  | Sphingolipid metabolism | 2.02 | 2.58E-03 |
| Profile 40 | 1060 | Citrate cycle (TCA cycle) | 7.08 | 3.61E-16 |
|  |  | Propanoate metabolism | 4.42 | 9.43E-08 |
|  |  | ABC transporters | 5.31 | 5.90E-08 |
|  |  | Tryptophan metabolism | 2.95 | 8.99E-08 |
|  |  | Alanine, aspartate and glutamate metabolism | 4.42 | 7.48E-07 |
|  |  | Pyruvate metabolism | 5.90 | 3.284E-06 |
|  |  | Fatty acid biosynthesis | 3.54 | 4.94E-06 |
|  |  | beta-Alanine metabolism | 2.65 | 1.60E-05 |
|  |  | Lysine degradation | 2.36 | 1.75E-04 |
|  |  | Valine, leucine and isoleucine degradation | 2.36 | 3.90E-04 |
|  |  | Purine metabolism | 7.67 | 5.18E-04 |
|  |  | Glycine, serine and threonine metabolism | 3.24 | 1.36E-03 |
|  |  | Phenylalanine, tyrosine and tryptophan biosynthesis | 2.06 | 1.51E-03 |
|  |  | Pantothenate and CoA biosynthesis | 2.06 | 2.99E-03 |
|  |  | Synthesis and degradation of ketone bodies | 0.88 | 3.37E-03 |
|  |  | Thiamine metabolism | 1.47 | 4.21E-03 |
|  |  | Biosynthesis of unsaturated fatty acids | 1.47 | 5.29E-03 |
|  |  | Glycolysis / Gluconeogenesis | 3.54 | 5.64E-03 |
|  |  | Peroxisome | 3.24 | 8.86E-03 |
| Profile 43 | 976 | Biosynthesis of terpenoids and steroids | 1.24 | 5.01E-04 |
|  |  | Selenocompound metabolism | 3.11 | 2.86E-04 |
|  |  | Carotenoid biosynthesis | 2.48 | 4.41E-03 |
|  |  | Fatty acid elongation | 1.86 | 8.74E-03 |
| Profile 46 | 809 | Nitrogen metabolism | 2.71 | 1.43E-04 |
|  |  | Aminoacyl-tRNA biosynthesis | 3.62 | 8.25E-03 |
| Profile 49 | 2062 | Fatty acid elongation | 3.43 | 1.41E-12 |
|  |  | Fatty acid biosynthesis | 3.68 | 1.84E-07 |
|  |  | Amino sugar and nucleotide sugar metabolism | 3.68 | 6.75E-06 |
|  |  | Fructose and mannose metabolism | 2.45 | 1.74E-04 |
|  |  | Protein processing in endoplasmic reticulum | 5.39 | 5.78E-04 |
|  |  | Fatty acid degradation | 1.96 | 1.60E-03 |
|  |  | Cutin, suberine and wax biosynthesis | 0.74 | 1.35E-03 |
|  |  | Ascorbate and aldarate metabolism | 1.47 | 1.57E-03 |
|  |  | MAPK signaling pathway - plant | 2.45 | 3.23E-03 |
|  |  | Protein export | 1.96 | 3.83E-03 |
|  |  | Plant hormone signal transduction | 1.72 | 4.25E-03 |
|  |  | Prodigiosin biosynthesis | 0.74 | 6.75E-03 |
